# Follow-up care over 12 months of patients with prostate cancer in Spain: A multicenter prospective cohort study

**DOI:** 10.1097/MD.0000000000027801

**Published:** 2021-11-24

**Authors:** Xavier Bonfill, María José Martinez-Zapata, Robin WM Vernooij, María José Sánchez, María Morales-Suárez-Varela, José Ignacio Emparanza, Montse Ferrer, José Ignacio Pijoan, Joan Palou, Eva Madrid, Víctor Abraira, Javier Zamora

**Affiliations:** a CIBER de Epidemiología y Salud Pública (CIBERESP), Barcelona, Spain; b Iberoamerican Cochrane Centre, Institute of Biomedical Research Sant Pau (IIB Sant Pau), Barcelona, Spain; c Public Health and Clinical Epidemiology Service, Hospital de la Santa Creu i Sant Pau, Spain; d Universitat Autònoma de Barcelona, Barcelona, Spain; e Cochrane Ecuador, Centro de Investigación en Salud Pública y Epidemiología Clínica (CISPEC), Facultad de Ciencias de la Salud Eugenio Espejo, Universidad Tecnológica Equinoccial, Quito, Ecuador; f Escuela Andaluza de Salud Pública, Instituto de Investigación Biosanitaria de Granada, Barcelona, Spain; g Unit of Public Health and Environmental Care, Department of Preventive Medicine, University of Valencia, Valencia, Spain; h Clinical Epidemiology Unit, Hospital Universitario Donostia, BioDonostia, San Sebastian, Spain; i Health Services Research Group, IMIM (Hospital del Mar Medical Research Institute), Barcelona, Spain; j Clinical Epidemiology Unit, Hospital, Universitario Cruces, Biocruces, Barakaldo, Spain; k Fundació Puigvert, Barcelona, Spain; l Cochrane Centre Universidad de Valparaíso, Chile, Interdisciplinary Centre for Health Studies CIESAL, Department of Public Health - School of Medicine - Universidad de Valparaíso, Chile; m Unidad de Bioestadística Clínica, Hospital Universitario Ramón y Cajal, IRYCIS, Madrid, Spain; n Barts and the London School of Medicine and Dentistry, Queen Mary University London, London, UK.

**Keywords:** hormone therapy, multicenter study, multivariate analysis, prostate cancer, prostatic neoplasms, radiotherapy, surgery

## Abstract

The therapeutic approach is crucial to prostate cancer prognosis. We describe treatments and outcomes for a Spanish cohort of patients with prostate cancer during the first 12 months after diagnosis and identify the factors that influenced the treatment they received.

This multicenter prospective cohort study included patients with prostate cancer followed up for 12 months after diagnosis. Treatment was stratified by factors such as hospital, age group (<70 and ≥70 years), and D’Amico cancer risk classification. The outcomes were Eastern Cooperative Oncology Group (ECOG) performance status, adverse events (AEs), and mortality. The patient characteristics associated with the different treatment modalities were analyzed using multivariate logistic regression.

We included 470 men from 7 Spanish tertiary hospitals (mean (standard deviation) age 67.8 (7.6) years), 373 (79.4%) of which received treatment (alone or in combination) as follows: surgery (n = 163; 34.7%); radiotherapy (RT) (n = 149; 31.7%); and hormone therapy (HT) (n = 142; 30.2%). The remaining patients (n = 97) were allocated to no treatment, that is, watchful waiting (14.0%) or active surveillance (5.7%). HT was the most frequently administered treatment during follow-up and RT plus HT was the most common therapeutic combination. Surgery was more frequent in patients aged <70, with lower histologic tumor grades, Gleason scores <7, and lower prostate-specific antigen levels; while RT was more frequent in patients aged ≥70 with histologic tumor grade 4, and higher ECOG scores. HT was more frequent in patients aged ≥70, with histologic tumor grades 3 to 4, Gleason score ≥8, ECOG ≥1, and higher prostate-specific antigen levels. The number of fully active patients (ECOG score 0) decreased significantly during follow-up, from 75.3% at diagnosis to 65.1% at 12 months (*P* < .001); 230 (48.9%) patients had at least 1 AE, and 12 (2.6%) patients died.

Surgery or RT were the main curative options. A fifth of the patients received no treatment. Palliative HT was more frequently administered to older patients with higher tumor grades and higher Gleason scores. Close to half of the patients experienced an AE related to their treatment.

## 1 Introduction

Prostate cancer is the most common cancer in men in both Western Europe (over lung cancer, in second place) and in Spain, with an estimated incidence of 171.4 and 147.9 patients per 100,000 men per year, respectively.^[1]^ However, mortality for prostate cancer is relatively low in comparison with other malignancies. Prognosis depends not only on patient characteristics, but also on the ability of healthcare systems to timely detect and treat patients with prostate cancer.

Once diagnosed, patients are generally treated according to their performance status, clinical cancer stage, tumor characteristics, and—ideally—individual values and preferences. In addition to making recommendations to guide clinical management of prostate cancer patients,^[2,3]^ it is important to conduct studies that focus on how patients are treated in the real world and the association between their characteristics and the specific treatment modalities. Some initiatives have been recently published in this regard, such as an international prospective cohort study that reported the characteristics of cancer patients and toxicity related to radiotherapy (RT).^[4]^ Another international initiative has been developed in Asia in a cohort of patients with advanced prostate cancer.^[5]^

We also published the baseline clinical characteristics, diagnoses, and factors affecting patient care intervals for a prospective cohort study of the incidence of prostate cancer in Spain.^[6]^ The current article reports the results of the first 12-month follow-up after diagnosis of this cohort of patients; we describe the treatments and outcomes, and identify the factors that influenced the therapy received during this follow-up period.

## 2 Methods

We conducted a multicenter observational cohort study on patients with prostate cancer attending 7 tertiary hospitals in Spain: Hospital Universitario 12 de Octubre (Hospital A); Hospital Universitario Ramón y Cajal (Hospital B) in Madrid; Hospital Universitario Donostia (Hospital C) in Donostia-San Sebastián; Hospital General Universitario de Valencia (Hospital D) in Valencia; Hospital Universitario Virgen de las Nieves (Hospital E) in Granada; Fundació Puigvert-Hospital de la Santa Creu i Sant Pau (Hospital G) (co-ordinating center); and Hospital del Mar (Hospital F) in Barcelona. The protocol was approved by all centers’ Research Ethics Committees.

Patients with prostate cancer were consecutively enrolled from October 2010 to September 2011. Inclusion criteria were patients with histologically proven and newly diagnosed prostate cancer at any stage of the disease, who were being treated at any of the 7 participating hospitals, and who had provided their informed consent.

We collected the following data: age; body mass index; World Health Organization (WHO) histologic tumor grade (1–4)^[7]^; prostate-specific antigen (PSA) value at diagnosis; total Gleason score^[8]^; clinical cancer stage (I–IV); tumor stage (T(Tumor size) N (lymph Nodes ) M (Metastasis) Classification of Malignant Tumors Staging System)^[9]^; intervention, that is surgery, RT, hormone therapy (HT), or watchful waiting/active surveillance (defined as no treatment other than diagnostic tests such as rectal examination, prostatic ultrasound, biopsy, or PSA measurement); Eastern Cooperative Oncology Group (ECOG) performance status score; adverse events (AEs) based in the Common Terminology Criteria for Adverse Events,^[10]^ and mortality during follow-up.

Using TNM tumor stages, PSA values, and Gleason scores, we classified patients into the following D’Amico risk^[11]^ groups: low risk (PSA 10 ng/mL, Gleason ≤6, and T1c-T2a); intermediate risk (PSA 10–20 ng/mL, Gleason 7, and T2b); and high risk (PSA >20 ng/mL, Gleason ≥8, and T2c-T3a). We stratified the analysis by age (<70 years and ≥70 years), initial treatment, D’Amico risk, hospital, and ECOG score.

We established the cut-off age at 70 years based on a national male life expectancy of 80 years,^[12]^ and the fact that clinical treatment guideline recommendations depend on whether the patient's life expectancy is more or less than 10 years at the time of diagnosis.^[13]^

For the descriptive analyses, we used relative frequencies for categorical variables, and either mean and standard deviation or median and interquartile range for continuous variables, depending on the skewness of the data distribution.

The proportion of missing values for each variable is reported. To study baseline patient characteristics associated with specific treatment modalities, we used 3 independent multivariate logistic regression models. Each model had a binary outcome measure representing whether a patient received as first-line treatment the modalities: surgery (model 1), RT (model 2), or hormonal therapy (model 3). We performed a backward elimination strategy to fit the most final parsimonious model containing the clinical and demographic factors significantly associated to the odds of receiving each one of the treatment alternatives. At each iteration, we excluded a variable from the model when its *P* value was greater than .05, excluding first the variable with higher *P* value. The potential predictors included in the maximal model were selected based on clinical plausibility, and included age, body mass index, WHO histological tumor grade, TNM tumor stage, total Gleason score, and PSA value at diagnosis. We report the odds ratio and the corresponding 95% confidence interval. We used the non-parametric Friedman test for repeated measures to estimate ECOG within-patient change across follow-up assessments (i.e., baseline, 6 months, and 12 months). This analysis was performed on the overall cohort and stratified by received treatment modality (i.e., surgery, RT, and HT).

Data were analyzed using SPSS statistical software version 20.0 (SPSS Inc., Chicago, IL) and Stata v12 software (StataCorp. 2011 Stata Statistical Software: Release 12. StataCorp LP, College Station, TX).

## 3 Results

**Figure 1:**
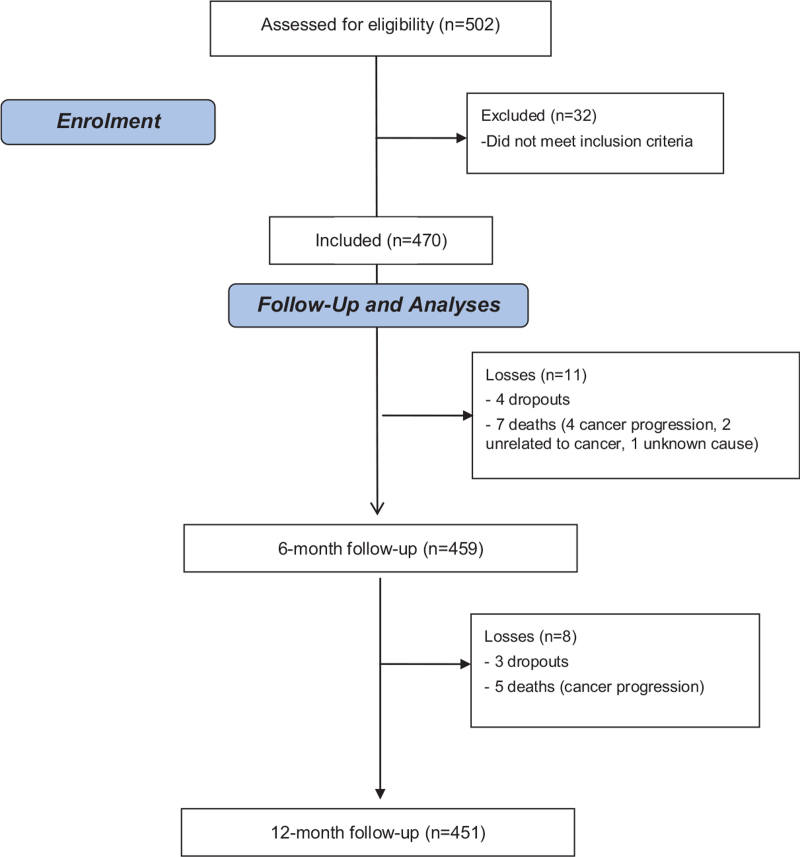
Patient flow chart.

In total, we recruited 470 patients: 451 (96.0%) of them completed the 12-month follow-up, 12 (2.6%) died, and 7 dropped out of the study (Fig. 1). The cause of death for 4 patients was prostate cancer progression; 2 died from other cause unrelated to cancer; and the cause was unknown for 1 patient. Mean (standard deviation) age was 67.8 (7.6) years, and 277 (58.9%) patients were under 70 years old (Table 1).

**Table 1:** Descriptive characteristics of patients by baseline treatment.

	All patients N = 470 n (%)	Surgery N = 163 n (%)	Radiotherapy N = 149 n (%)	Hormone therapy N = 142 n (%)	Watchful waiting N = 97 n (%)
Age					
<70 yrs	277 (58.9)	136 (83.4)	81 (54.4)	55 (38.7)	51 (52.6)
≥70 yrs	182 (38.7)	25 (15.3)	67 (45.0)	82 (57.7)	42 (43.3)
Missing data	11 (2.3)	2 (1.2)	1 (0.7)	5 (3.5)	4 (4.1)
BMI					
<25	95 (20.2)	32 (19.8)	25 (16.7)	27 (19.0)	27 (27.8)
≥25–<30	232 (49.4)	89 (54.6)	69 (46.3)	65 (47.8)	43 (44.3)
≥30	125 (26.6)	36 (22.1)	49 (32.9)	46 (32.4)	23 (23.7)
Missing data	18 (3.8)	6 (3.7)	6 (4.0)	4 (2.8)	4 (4.1)
D’Amico risk					
Low	169 (36.0)	66 (40.5)	61 (40.9)	26 (18.3)	33 (34.0)
Intermediate	114 (24.3)	53 (32.5)	32 (21.5)	27 (19.0)	23 (23.7)
High	186 (39.6)	44 (27.0)	55 (36.9)	88 (62.0)	41 (42.3)
Missing data	1 (0.1)	0	1 (0.7)	1 (0.7)	0
Histologic grade					
1	23 (4.9)	7 (4.3)	7 (4.7)	4 (2.8)	7 (7.2)
2	195 (41.5)	87 (53.4)	61 (40.9)	46 (32.4)	31 (32.0)
3/4	169 (36.0)	45 (27.6)	49 (32.9)	75 (52.8)	41 (42.3)
Missing data	81 (17.7)	24 (14.7)	32 (21.5)	17 (11.9)	18 (18.6)
Tumor stage					
Tx	1 (0.2)	0	0	1 (0.7)	0
T1a-c	193 (41.1)	70 (42.9)	64 (43.0)	38 (26.8)	41 (42.3)
T2a-c	188 (40.0)	73 (44.8)	54 (36.2)	55 (38.7)	41 (42.3)
T3a-b	74 (15.7)	16 (9.8)	28 (18.8)	41 (28.9)	12 (12.4)
T4	8 (1.7)	0	0	5 (3.5)	2 (2.1)
Missing data	6 (1.3)	4 (2.5)	3 (2.0)	2 (1.4)	1 (1.0)
Gleason grade					
1 (≤6)	261 (55.1)	103 (63.2)	88 (59.1)	54 (38.0)	50 (51.5)
2 (7 = 3+4)	98 (20.9)	42 (25,8)	30 (20.1)	24 (16.9)	21 (21.6)
3 (7 = 4+3)	28 (6.1)	9 (5.6)	9 (6.0)	15 (10.6)	4 (4.1)
4 (8)	42 (8.9)	3 (1.8)	14 (9.4)	24 (16.9)	12 (12.4)
5 (9, 10)	32 (6.8)	3 (1.8)	5 (3.4)	19 (13.4)	10 (10.3)
Missing data	9 (1.9)	3 (1.8)	3 (2.0)	6 (4.2)	0
PSA					
Median (IQR)	7.6 (7.8)	6.5 (4.4)	7.8 (7.8)	13.0 (14.4)	6.7 (5.2)

Eighty-seven patients received a combination of treatments. BMI = body mass index, IQR = interquartile range, PSA = prostate-specific antigen.

The most frequent WHO histological grades were 2 (n = 195; 41.5%) and 3/4 (n = 169; 36.0%). Most patients (62.5%) had T1b-T2b tumors. Over half of the patients (55.1%) scored ≤6 on the Gleason scale, 26.8% scored 7, and 15.7% scored ≥8 (Table 1).

After diagnosis, 97 patients (20.6%) were allocated to watchful waiting and 373 (79.4%) were treated as follows: surgery (n = 163; 34.7%); RT (n = 149; 31.7%); and HT (n = 142; 30.2%) (Table 1). Most patients treated with surgery were <70 years (83.4%) and had T1b-T2b tumor stage (66.3%). Among the patients treated with RT, 45% were ≥70 years, 67.1% had T1b-T2b tumor stage, and 12.8% had a Gleason score ≥8. Patients treated with HT were generally ≥70 years (57.7%), had higher Gleason scores (≥8; 30.3%); around half each had T1b-T2b and T2c-T4 tumor stages (50.7% and 46.5%, respectively) (Table 1).

Just under one fifth of patients (n = 87; 18.5%) received a combination of treatments, most frequently RT plus HT (n = 72; 15.3%) (Table 2). During the follow-up period, 165 patients (35.1%) underwent surgery, 164 (34.9%) received RT, and 193 (41.1%) received HT. By the end of this period, 27 patients (5.7%) allocated to watchful waiting at baseline were reallocated to active surveillance, and 66 (14.1%) continued in watchful waiting (Table 2).

**Table 2:** Treatment distribution at baseline per patient and during 12-month follow-up (N = 470 patients).

Treatment	Baseline n (%)	Follow-up^a^ n (%)
Surgery	151 (32.1)	3 (0.6)
Radiotherapy	71 (15.1)	15 (3.2)
Hormone therapy	66 (14.1)	49 (10.4)
Chemotherapy	0	3 (0.6)
Other	2 (0.4)	6 (1.3)
Surgery + radiotherapy	7 (1.5)	0
Surgery + hormone therapy	5 (1.1)	0
Radiotherapy + hormone therapy	71 (15.1)	2 (0.4)
Watchful waiting	97 (20.6)	66 (14.1)
Active surveillance	0	27 (5.7)
Only baseline treatment	–	299 (63.7)

a Patients with new treatments during follow-up.

**Figure 2:**
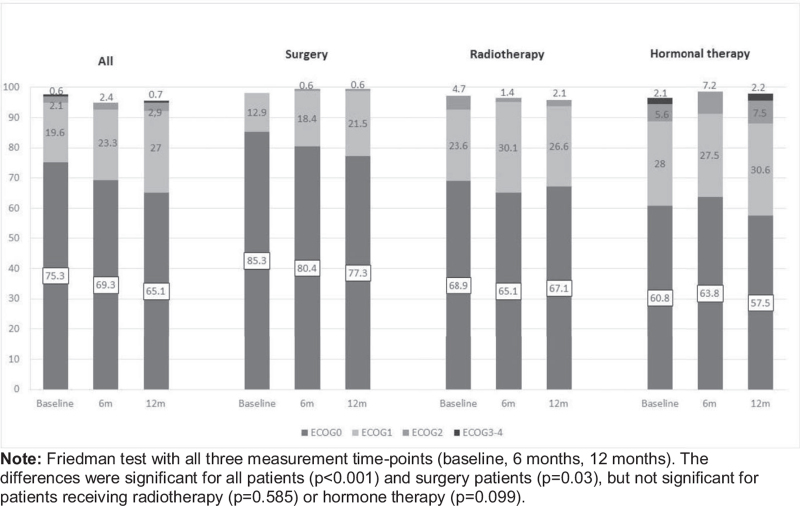
Eastern Cooperative Oncology Group (ECOG) scores by study time-points and treatments.

While 75.3% of the patients had an ECOG score of 0 at baseline (Fig. 2), this percentage was observed to fall over time, with 69.3% scoring 0 at 6 months, and 65.1% scoring 0 at 12 months.

Among the patients <70 years undergoing surgery, 41.2%, 30.9%, and 27.9% had low, intermediate, and high D’Amico risk, respectively (*P* = .027); and among those receiving HT, 18.2%, 20.2%, and 61.8% had low, intermediate, and high risk, respectively (*P* < .001). In this age group there was no difference in the odds of patients receiving RT (*P* = .098) or being assigned to watchful waiting (0.748) (Table 3). Among the patients ≥70 years undergoing surgery, 32.0%, 44.0%, and 24.0% had low, intermediate, and high D’Amico risk, respectively (*P* = .044); among those receiving RT, 36.4%, 28.8%, and 34.8% had low, intermediate, and high risk, respectively (*P* = .030); and among those receiving HT, 17.3%, 18.5%, and 64.2% had low, intermediate, and high risk, respectively (*P* < .001). In this age group, there was no difference in the odds of patients being assigned to watchful waiting (0.756) (Table 3).

**Table 3:** D’Amico risk stratified by age and treatment at baseline.

	<70 yrs N = 277				≥70 yrs N = 182			
D’Amico risk	Low N = 115 n (%)	Intermediate N = 68 n (%)	High N = 94 n (%)	*P*	Low N = 49 n (%)	Intermediate N = 46 n (%)	High N = 86 n (%)	*P*
Surgery	56 (41.2)	42 (30.9)	38 (27.9)	.027	8 (32.0)	11 (44.0)	6 (24.0)	.044
Radiotherapy	36 (44.4)	13 (16.1)	32 (39.5)	.098	24 (36.4)	19 (28.8)	23 (34.8)	.030
Hormone therapy	10 (18.2)	11 (20.2)	34 (61.8)	<.001	14 (17.3)	15 (18.5)	52 (64.2)	<.001
Watchful waiting	23 (46.0)	12 (24.0)	15 (30.0)	.748	10 (23.3)	11 (25.6)	22 (51.2)	.756

Patients could receive more than 1 treatment. Percentages are calculated for rows. Data for 11 cases were missing for D’Amico risk.

At baseline, the first-line treatments for patients with prostate cancer were surgery for hospitals C (51.4%), F (63.6%), and G (50.7%); RT for hospitals A (22.9%) and B (38.5%); and HT for hospitals D (42.3%) and E (50.0%). HT was the most prescribed treatment in the 12-month follow-up, although its use ranged from 5.5% in hospital B to 50% in hospital E.

The multivariate logistic regression analysis (Table 4) revealed that patients <70 years, with low tumor histologic grades, Gleason scores <7, and low PSA levels were more likely to undergo surgery as first-line treatment for prostate cancer. Patients with a histologic tumor grade 4 and higher ECOG scores were more likely to be treated with RT. Lastly, patients ≥70 years, with tumor grades 3 or 4, Gleason scores ≥8, ECOG scores ≥1, and higher PSA levels were most likely to be treated with HT.

**Table 4:** Multivariate logistic regression: baseline patient characteristics associated with treatment modalities.

	Surgery		Radiotherapy		Hormone therapy	
	OR (CI 95%)	*P*	OR (CI 95%)	*P*	OR (CI 95%)	*P*
Age (yrs)						
<70	1 (Reference)				1 (Reference)	
≥70	0.22 (0.13–0.38)	<.001			2.17 (1.27–3.72)	.005
Histologic grade						
≤2	1 (Reference)		1 (Reference)			
3	1.08 (0.49–2.35)	.851	0.84 (0.53–1.32)	.450		
4	0.21 (0.09–0.51)	.001	1.90 (1.03–3.50)	.040		
Gleason score						
≤6	1 (Reference)				1 (Reference)	
7	1.08 (0.49–2.35)	.842			1.02 (0.57–1.83)	.938
≥8	0.08 (0.01–0.43)	.003			4.24 (1.86–9.73)	.001
Log (PSA)	0.53 (0.36–0.77)	.001			3.17 (2.07–4.84)	<.001
ECOG score						
0			1 (Reference)		1 (Reference)	
1			1.41 (0.85–2.32)	.178	1.94 (1.06–3.56)	.033
≥2			3.05 (0.99–9.37)	.052	10.98 (1.91–63.1)	.007

CI = confidence interval, ECOG = Eastern Cooperative Oncology Group, OR = odds ratio, PSA = prostate-specific antigen.

A total of 230 AEs were reported, with 48.9% of patients experiencing at least one (Table 5). By intervention, the most frequent AEs were urinary incontinence (50.3%) and impotence (45.4%) for surgery; impotence (12.8%) and cystitis (12.8%) for RT; and hot flushes (26.1%), impotence (15.5), and reduced libido (14.8%) for HT. During the 12-month follow-up, 12 (2.6%) patients died.

**Table 5:** Adverse events by treatment through the 12-month follow-up period.

Surgery N = 163 n (%)		Radiotherapy N = 149 n (%)		Hormone therapy N = 142 n (%)	
Urinary incontinence	82 (50.3)	Impotence	19 (12.8)	Hot flushes	37 (26.1)
Impotence	74 (45.4)	Cystitis	19 (12.8)	Impotence	22 (15.5)
Urethral stricture	5 (3.1)	Intestinal alteration	10 (6.7)	Reduced libido	21 (14.8)
Fecal incontinence	2 (1.2)	Urinary incontinence	8 (5.4)	Diarrhea	6 (4.2)
Other	10 (6.1)	Proctitis	8 (5.4)	Rash	5 (3.5)
		Enteritis	5 (3.4)	Gynecomastia	4 (2.8)
		Others	21 (14.1)	Diarrhea	3 (2.1)
				Acne	2 (1.4)
				Breast tenderness	2 (1.4)
				Pruritus	2 (1.4)
				Osteoporosis	1 (0.7)
				Other	12 (8.5)
≥1 adverse effect	113 (69.3)		61 (40.9)		56 (39.4)

## 4 Discussion

This prospective cohort study describes healthcare practices at 7 Spanish hospitals for newly diagnosed prostate cancer patients, focusing on primary therapy and patient-relevant clinical outcomes observed within the first 12 months since diagnosis. The majority of the 470 included patients were under 70 years old and 81% had localized prostate cancer. Primarily, surgery or RT were the initial treatment modalities for 1 in 3 patients, as per guideline recommendations.^[3]^ HT was also administered in one third of patients as palliative treatment. During the 12-month follow-up, HT was the most frequent treatment and RT plus HT was the most frequent treatment combination.

One fifth of patients was assigned to watchful waiting after diagnosis, a similar rate to that reported by Hoffman et al^[13]^ in a 6-month follow-up study. Our watchful waiting percentage decreased to 14% at 12-month follow-up, as the remaining 5.7% were moved to active surveillance. In contrast, Hoffman et al^[13]^ described that more patients were in active surveillance at 18-month follow-up, while fewer than 2% remained in watchful waiting.

In our study a high proportion of patients with T1b-T2b tumor stage underwent surgery and received either RT or HT. A lower-than-expected proportion of patients with T2c-T4 tumor stage received RT or HT.

In agreement with guideline recommendations,^[3]^ surgery was more frequent in younger patients (<70 years) with low or intermediate cancer risk (D’Amico classification), whereas HT was more frequent in high-risk cases and older patients (≥70 years). However, RT administration was only influenced significantly by the risk classification in older patients and those with higher histological grade.

According to a recently published clinical trial, there is no single best treatment option for localized prostate cancer, as overall survival is similar for patients undergoing radical prostatectomy, RT, or active surveillance.^[14]^ However, we found that certain factors influenced decision-making regarding different therapeutic options: patients who underwent surgery were younger and had better prognostic factors (lower histologic tumor grades, Gleason scores, and PSA levels); patients treated with RT had higher histological tumor grades and poorer ECOG scores; and lastly, patients who received palliative HT were older and had poorer prognostic factors (more advanced tumors, higher Gleason scores, higher PSA levels, and higher ECOG scores).

Other cohort studies have been published recently, but their results are not completely comparable to our study. These studies included prevalent and incident patients,^[4,5]^ and our study only focused on incident prostate cancer patients. One international multicenter cohort study focused on patients receiving RT.^[4]^ Twenty-seven percent of included patients underwent a prostatectomy before RT, whereas in our study the proportion was only 1.5%. The proportion of patients receiving RT and HT also differed between studies—69% and 15%, respectively. Another international multicenter cohort study included advanced prostate cancer, whereas our study mainly included localized prostate cancer.^[5]^

While the Spanish National Health System is a public system providing universal coverage and free-of-charge treatments to patients, we found important differences in therapeutic choices between the 7 participating hospitals, with some preferring surgery, whereas others preferred RT or HT. This variability was probably related to patient characteristics and differing hospital criteria regarding treatments.

At baseline, 3 quarters of patients had a good performance status that worsened over the follow-up period. Only a small proportion of patients (2.6%) died during the first year, mainly due to cancer-related reasons. The most common AEs match those reported in previous studies,^[14,15]^ such as urinary incontinence and impotence for surgery, impotence^[4]^ and cystitis for RT, and hot flushes for HT.

Regarding limitations, our study may be affected by potential information bias, given that our data were prospectively obtained from hospital records and participants. However, we consider this limitation of little actual relevance.

A main strength of our study is that our patient sample is probably representative of the annual incident cases of patients diagnosed with prostate cancer in Spain, since they were recruited in 7 hospitals located in 5 different regions. In addition, the prospective nature of the study guarantees greater data consistency and accuracy, and so overcomes the typical shortcoming of retrospective data collection affecting similar studies carried out elsewhere. The relatively small number of patients lost to follow-up (4.2%) reinforces the validity of our results. A longer follow-up will undoubtedly be useful in further assessing the impact of diagnosis and therapy on prostate cancer patients.

## 5 Conclusion

Surgery and RT were the most common curative options used on initial diagnosis of prostate cancer. Watchful waiting was applied to 1 in 5 patients after diagnosis. Palliative HT was the most prescribed follow-up treatment. Surgery was more frequently indicated in younger patients with better prognostic factors. HT was more frequent in older patients, with more advanced tumor stages and higher Gleason scores. Around half of the patients experienced an AE related to the treatment. Performance status decreased steadily in the first year after diagnosis. The treatments administered by the participating hospitals varied widely.

## Acknowledgments

Ailish Maher and Andrea Cervera Alepuz revised the English in a version of this manuscript. Maria José Martinez Zapata is funded by a Miguel Servet research contract (CPII20/00023).

**EMPARO Study Group: Coordinating investigator:** Xavier Bonfill Cosp (Iberoamerican Cochrane Centre, Public Health and Clinical Epidemiology Service, Hospital de la Santa Creu i Sant Pau, IIB Sant Pau, Barcelona, Spain).

**Project manager:** María José Martínez Zapata (Iberoamerican Cochrane Centre, IIBSant Pau, Barcelona, Spain).

**Clinical research assistants:** Alborada Martínez (Universidad de Valencia); Enrique Morales Olivera (Escuela Andaluza de Salud Pública, Granada, Spain); Esther Canovas, Laura Muñoz, Gemma Mas, René Acosta, Ekaterina Popova (Iberoamerican Cochrane Centre, IIB Sant Pau, Barcelona, Spain); Irma Ospina (Hospital 12 de Octubre, Madrid, Spain); María José Velázquez (Hospital Donostia, Donostia, Spain); Tamara Ruiz Merlo (Hospital Ramón y Cajal, Madrid, Spain); Gael Combarros Herman, Judit Tirado Muñoz (IMIM-Hospital del Mar Medical Research Institute, Barcelona, Spain).

**Statistical analysis:** Robin W.M. Vernooij (Iberoamerican Cochrane Centre, IIB Sant Pau, Barcelona, Spain); Javier Zamora and Claudia Coscia Requena (Hospital Ramón y Cajal, Madrid, Spain).

**Co-investigators: Barcelona, Spain**

Albert Frances (Hospital del Mar); Carola Orrego Villagran, Rosa Suñol (Instituto Universitario Avedis Donabedian); Dimelza Osorio, Gemma Sancho Pardo, Ignasi Bolívar, José Pablo Maroto, María Jesús Quintana, Cristina Martin (Hospital de la Santa Creu i Sant Pau); Ferran Algaba, Palou Redorta, Salvador Esquena (Fundació Puigvert); Jordi Bachs (Fundació Privada Hospital de la Santa Creu i Sant Pau); María José Martínez Zapata (Iberoamerican Cochrane Centre, IIB Sant Pau); Montserrat Ferrer Fores, Stefanie Schmidt, Olatz Garin, Virginia Becerra Bachito, Yolanda Pardo (IMIM-Hospital del Mar Medical Research Institute).

**Bilbao, Spain**

Amaia Martínez Galarza, José Ignacio Pijoán Zubizarreta (Hospital Universitario Cruces/BioCruces Health Research Institute).

**Granada, Spain**

Armando Suárez Pacheco, Cesar García López, José Manuel Cozar Olmo (Hospital Universitario Virgen de las Nieves); Carmen Martínez, Daysy Chang Chan, María José Sánchez Pérez (Escuela Andaluza de Salud Pública).

**Madrid, Spain**

Ana Isabel Díaz Moratinos, Angel Montero Luis, Asunción Hervás, Carmen Vallejo Ocaña, Costantino Varona, Javier Burgos, Javier Zamora, Jose Alfredo Polo Rubio, Luis López-Fando Lavalle, Miguel Angel Jimenez Cidre, Muriel García, Alfonso, Nieves Plana Farras, Rosa Morera Lopez, Sonsoles Sancho Garcia, Victor Abraira, Victoria Gomez Dos Santos (Hospital Ramón y Cajal); Agustín Gómez de la Cámara, Javier de la Cruz, Juan Passas Martínez, Humberto García Muñoz, María Ángeles Cabeza Rodríguez (Hospital 12 de Octubre).

**San Sebastián, Spain**

Irune Ruiz Díaz, José Ignacio Emparanza, Juan Pablo Sanz Jaka (Hospital Universitario Donostia).

**Valencia, Spain**

Agustín Llopis González, María Morales (Universidad de Valencia); Carlos Camps, Cristina Caballero Díaz, Emilio Marqués Vidal, Francisco Sánchez Ballester, Joaquín Ulises Juan Escudero, Jorge Pastor Peidro, José López Torrecilla, María Macarena Ramos Campos, Miguel Martorell Cebollada (Consorcio Hospital General Universitario de Valencia).

## Author contributions

**Conceptualization:** Xavier Bonfill.

**Data curation:** Maria Jose Martinez-Zapata.

**Formal analysis:** Robin WM Vernooij, Víctor Abraira, Javier Zamora.

**Funding acquisition:** Xavier Bonfill, Javier Zamora.

**Investigation:** Xavier Bonfill, Maria Jose Martinez-Zapata, María José Sánchez, María Morales-Suárez-Varela, José Ignacio Emparanza, Montse Ferrer, José Ignacio Pijoan, Joan Palou.

**Methodology:** Xavier Bonfill, Maria Jose Martinez-Zapata, Eva Madrid, Víctor Abraira, Javier Zamora.

**Supervision:** Xavier Bonfill, Maria Jose Martinez-Zapata.

**Validation:** Maria Jose Martinez-Zapata, Robin WM Vernooij.

**Writing – original draft:** Xavier Bonfill, Maria Jose Martinez-Zapata, Robin WM Vernooij, María Morales-Suárez-Varela, Eva Madrid, Javier Zamora.

**Writing – review & editing:** Xavier Bonfill, Maria Jose Martinez-Zapata, Robin WM Vernooij, María José Sánchez, María Morales-Suárez-Varela, José Ignacio Emparanza, Montse Ferrer, José Ignacio Pijoan, Joan Palou, Eva Madrid, Víctor Abraira, Javier Zamora.

## References

[1] European Cancer Information System (ECIS). © European Union, 2018. Available at: https://ecis.jrc.ec.europa.eu. Accessed June 23, 2019.

[2] European Association Urology (EAU) guidelines. Edn. Presented at the EAU Annual Congress Copenhagen 2018. ISBN 978-94-92671-01-1.

[3] Sanda MG Cadeddu JA Kirkby E. Clinically localized prostate cancer: AUA/ASTRO/SUO guideline. Part I: risk stratification, shared decision making, and care options. J Urol 2018;199:683–90. 29203269 10.1016/j.juro.2017.11.095

[4] Seibold P Webb A Aguado-Barrera ME. REQUITE: a prospective multicentre cohort study of patients undergoing radiotherapy for breast, lung or prostate cancer. Radiother Oncol 2019;138:59–67. 31146072 10.1016/j.radonc.2019.04.034

[5] Uemura H Ye D Kanesvaran R. United in Fight against prOstate cancer (UFO) registry: first results from a large, multi-centre, prospective, longitudinal cohort study of advanced prostate cancer in Asia. BJU Int 2020;125:541–52. 31868997 10.1111/bju.14980 PMC7187217

[6] Bonfill X Martinez-Zapata MJ Vernooij RW. Clinical intervals and diagnostic characteristics in a cohort of prostate cancer patients in Spain: a multicentre observational study. BMC Urol 2015;15:60. 26134117 10.1186/s12894-015-0058-x PMC4488131

[7] Eble JN, Sauter G, Epstein JI, et al (Eds.). World Health Organization Classification of Tumours. Pathology and Genetics of Tumours of the Urinary System and Male Genital Organs. Lyon: IARC Press; 2004.

[8] Epstein JI Allsbrook WC Jr Amin MB Egevad LL. ISUP Grading Committee. The 2005 International Society of Urological Pathology (ISUP) consensus conference on Gleason grading of prostatic carcinoma. Am J Surg Pathol 2005;29:1228–42. 16096414 10.1097/01.pas.0000173646.99337.b1

[9] American Joint Committee on Cancer: AJCC Cancer Staging Manual. 6th ed. 2002;New York, NY: Springer, 335–340.

[10] Therapy evaluation program, common terminology criteria for adverse events, Version 4.0, DCTD, NCI, NIH, DHHS. June 3, 2010.

[11] D’Amico AV Whittington R Malkowicz SB. Biochemical outcome after radical prostatectomy, external beam radiation therapy, or interstitial radiation therapy for clinically localized prostate cancer. JAMA 1998;280:969–74. 9749478 10.1001/jama.280.11.969

[12] Instituto Nacional de Estadística (INE), Spain 2018. Mujeres y hombres en España /Salud /4.1 Esperanza de vida. Available at: http://www.ine.es/ss/Satellite?c=INESeccion_C&p=1254735110672&pagename=ProductosYServicios%2FPYSLayout&cid=1259926380048&L=1. Accessed June 23, 2019.

[13] Hoffman KE Niu J Shen Y. Physician variation in management of low-risk prostate cancer: a population-based cohort study. JAMA Intern Med 2014;174:1450–9. 25023650 10.1001/jamainternmed.2014.3021 PMC4372187

[14] Hamdy FC Donovan JL Lane JA. 10-Year outcomes after monitoring, surgery, or radiotherapy for localized prostate cancer. N Engl J Med 2016;375:1415–24. 27626136 10.1056/NEJMoa1606220

[15] Wilt TJ Jones KM Barry MJ. Follow-up of prostatectomy versus observation for early prostate cancer. N Engl J Med 2017;377:132–42. 28700844 10.1056/NEJMoa1615869

